# Immunohistochemical approach to the pathogenesis of clinical cases of Bovine *Herpesvirus *type 5 infections

**DOI:** 10.1186/1746-1596-5-57

**Published:** 2010-09-10

**Authors:** Tereza C Cardoso, Heitor F Ferrari, Andrea F Garcia, Livia C Bregano, Alexandre L Andrade, Adriana HC Nogueira

**Affiliations:** 1 Department of DCCA and DCCRA, Veterinary School, Laboratory of Virology, Clovis Pestana Street, Araçatuba, 16.050-680, Brazil; 2 Laboratory of Animal Pathology, Clovis Pestana Street, Araçatuba, 16.050-680, Brazil; 3 Biologic Institute, Laboratory of bovine diseases, São Paulo, Brazil

## Abstract

Meningoencephalitis by *Herpesvirus *type 5 (BoHV-5) in cattle has some features that are similar to those of herpetic encephalitis in humans and other animal species. Human *Herpesvirus *3 (commonly known as Varicella-zoster virus 1), herpes simplex viruses (HSV), and equid *Herpesvirus *1 (EHV-1) induce an intense inflammatory, vascular and cellular response. In spite of the many reports describing the histological lesions associated with natural and experimental infections, the immunopathological mechanisms for the development of neurological disorder have not been established. A total of twenty calf brains were selected from the Veterinary School, University of São Paulo State, Araçatuba, Brazil, after confirmation of BoHV-5 infection by virus isolation as well as by a molecular approach. The first part of the study characterized the microscopic lesions associated with the brain areas in the central nervous system (CNS) that tested positive in a viral US9 gene hybridization assay. The frontal cortex (Fc), parietal cortex (Pc), thalamus (T) and mesencephalon (M) were studied. Secondly, distinct pathogenesis mechanisms that take place in acute cases were investigated by an immunohistochemistry assay. This study found the frontal cortex to be the main region where intense oxidative stress phenomena (AOP-1) and synaptic protein expression (SNAP-25) were closely related to inflammatory cuffs, satellitosis and gliosis, which represent the most frequently observed neurological lesions. Moreover, MMP-9 expression was shown to be localized in the leptomeninges, in the parenchyma and around mononuclear infiltrates (*p *< 0.0001). These data open a new perspective in understanding the role of the AOP-1, MMP-9 and SNAP-25 proteins in mediating BoHV-5 pathogenesis and the strategies of host-virus interaction in order to invade de CNS.

## Background

Bovine *Herpesvirus *type 5 (BoHV-5) is member of the family *Herpesviridae*, sub-family *Alphaherpesvirinae*, and is the primary etiological agent of non-suppurative meningoencephalitis, which is described as a cause of significant economic losses to beef cattle in Latin America [1]. Outbreaks of BoHV-5-associated encephalitis have been reported worldwide; however, most positive cases are located in South America [2,3]. In addition, a marked neurotropism that frequently leads to fatal disease has been shown to be caused by BoHV-5, and the olfactory bulb and frontal cortex have been described as sources of virus replication [4-6].

Lesions related to central nervous system (CNS) infection have been well-described in cattle [3], rabbits [7], sheep [8] and goats [4], mainly with regard to neuronal degeneration and inflammatory response. The distribution of BoHV-5 DNA during the acute and latent phases of infection in experimentally infected rabbits and calves has been determined by amplification of glycoprotein C gene using the polymerase chain reaction (PCR) from brain suspensions [6]. However, to improve the *in situ *detection of viral genes, an *in situ *PCR protocol was established and used to implicate the olfactory bulb of the infected bovine CSN as the target region in natural cases of BoHV-5 infections [9].

Most of the neuro-pathological patterns have been established to characterize the microscopic lesions [3]. In addition, these patterns are controversial due to the difficulty in isolating BoHV-5 from the respective cases and also the different stages of the disease: acute infection, latency and re-activation of virus infection [5,10].

Metalloproteinases (MMPs) are zinc-dependent proteases that are secreted by most cell types as proenzymes. MMPs are classified in terms of their substrate specificity into classes such as gelatinases, collagenases and stromeolysins [11]. MMPs play key roles in inflammatory responses through protease-release of cell membrane-bound factors and activation or inactivation of cytokines, chemokines and growth factors. MMP-9 is known to digest gelatins and types III, IV and V collagen [11]. Moreover, findings support a central role of MMP-9 in T-cell migration and in disruption of vascular basement membranes, and this protein has recently been shown to act directly in the blood-brain barrier after virus infection [12,13].

Mitochondria play an important role in the aerobic energy metabolism of living cells. The mitochondrial electron transport system consumes approximately 85% of the oxygen utilized by the cell, and about 5% of the oxygen is converted to reactive oxygen species (ROS). In the normal state of the cell, the adequate concentration of ROS participates in a wide variety of cellular functions, including cell proliferation, differentiation and apoptosis [14]. However, the significant induction of ROS or the depletion of cellular antioxidants induces cell death, and ROS are likely to act as signaling intermediates that are involved in the signal transduction mechanism for apoptosis. The anti-oxidant protein 1 (AOP-1) is an antioxidant protein and functions as a thioredoxin-dependent peroxidase, which scavenges ROS such as H_2_O_2 _[14].

Little information about cellular signaling and its possible participation in the pathogenesis of BoHV-5 infection in calves with neurological disorder is available. Also, other mechanisms that can trigger CSN damage must better established. To this end, we aimed to further investigate the BoHV-5-induced brain lesions associated with some pathogenic events, such as the expression of anti-oxidant protein 1 (AOP-1) and synaptosome-associated protein (SNAP-25) as well as the status of blood-brain barrier integrity (MMP-9) among 20 cattle brains. Secondly, the histological lesions were directly compared with the results of immunolabeling in the frontal cortex, parietal cortex, thalamus and mesencephalon.

## Materials and methods

### Samples and general procedures

For the present study, 20 cattle brains diagnosed as negative for rabies virus and positive for BoHV-5 were used [15]. These brains were obtained from routine necropsy at the veterinary school of the University of São Paulo State from 2004 to 2008 and had previously been confirmed as positive for BoHV-5 infection after virus isolation in the Madin-Darby bovine kidney (MDBK) cell culture system and molecular analysis [16]. The cases studied had shown different clinical signs at the ante-mortem inspection, as described previously [15]. The whole brain was sampled and dissected into four brain areas: the parietal cortex (Pc), frontal cortex (Fc), thalamus (T) and mesencephalon (M). Both hemispheres were analyzed in this study. The sections were immersed in 10% phosphate-buffered formalin, sectioned into 10- to 15-mm thick sections, blocked and immersed in 98% formic acid for 24 h and then embedded in paraffin wax. Sections (4-μm in thickness) were then obtained for routine Haematoxylin and Eosin (HE) staining, and the respective microscopic lesions were characterized at least by two different pathologists blind to the experimental design. In order to access the cross-reactive of antibodies, 25 BoHV-5 negative brain samples were used in this study for all analysis described followed.

### Immunohistochemistry assay (IHC)

To perform the IHC assay, a standard avidin-biotin-peroxidase complex (LSAB kit, Dako, CA, USA; code K0690-1) method was used as described previously [17]. Unstained sections (4-μm) were deparaffinized, rehydrated and washed in buffered saline with 0.1% Tween 80. The first step was to microwave the sections in citrate buffer (pH 6.1) for 15 min at 700 W to retrieve the viral antigen, which is normally damaged by formaldehyde fixation. Just before staining, the slides were treated three times with 2% (v/v) of hydrogen peroxide 30 vol diluted in 50% (v/v) methanol and deionized water for 30 min to inactivate the endogenous peroxidase. The slides were then washed 5 times for 10 min each wash in buffered saline to remove residues. The next step was to block nonspecific binding by incubating in 15% reconstituted dry nonfat milk for 90 min. The optimum primary antibody dilutions used are described in Table 1. The slides were covered by 200 μl of diluted antibody overnight at 4°C in a humidified chamber. Slides were washed in PBS, incubated with biotinylated secondary antibody (K0690 LSAB^+ ^Kit, Dako) for 45 min at room temperature. After 5 washes, 100 μl streptavidin-HRP complex (K0690 LSAB^+ ^Kit, Dako) was added to each slide, and the slides were incubated for 1 h at 37°C. In addition, a substrate made fresh in the dark, by mixing equal volumes of 0.02% hydrogen peroxide and 0.6 mg DAB (3,3- diaminobenzidine tetrahydrochloride, Gibco BRL, code 15972-011), was added to the slides for 30 min at room temperature. The reaction was stopped by washing with tap water and the specific brown color was revealed after counterstaining with Harry's hematoxylin. An intense brownish deposit was considered positive. Omission of the primary antibody was used as a negative control for the different antibodies. For a MMP-9 positive control, a canine mammary carcinoma histological section was used.

**Table 1:** Details of the primary antibodies used

	Antibody	Dilution	Species	Supplier
AOP-1	Anti-oxidant protein 1	1:1000	Mouse	Sigma-Aldrich^®^ (cat # A7674)
MMP-9	Metalloproteinase 9	1:1000	Mouse	Sigma-Aldrich^®^
				(cat # M5177)
SNAP-25	Synaptosome-associated 25kDa protein	1:10000	Mouse	Prestige Antibodies™ (cat # HPA001830)

### In situ hybridization assay (ISH)

To perform *in situ *hybridization, the DNA probe was prepared from PCR amplicons of BoHV-5 DNA US9 gene according to the description in a previous study [17]. After DNA purification from an agarose gel, the PCR product was linked into the TA vector (pGEMTEasy, Promega, Madison, WI, USA), and the ligation products were introduced into *E. coli *by heat shock. Positive colonies were confirmed by DNA sequencing. A confirmed positive colony was cultured, and its plasmid DNA was prepared using a commercially available kit (Promega Miniprep, Promega, Madison, WI, USA). The probe was generated by a PCR reaction targeting the gC gene located in the plasmid, as previously described, using a biotin-labeled reverse primer (Invitrogen™). Briefly, for the ISH assay, the slides from brain areas were submitted to the same protocol of de-waxing and re-hydration described earlier. The slides were then treated with proteinase *K *(10 μg/mL, Invitrogen™) for 10 min at room temperature and washed in PBS. A 259-bp denatured biotin-labeled probe was applied in a solution consisting of 2 μl (2 ng/ml) probe and 98 μl of pre-hybridization buffer (50% formamide, 5% bovine serum albumin, 1% N-lauroylsarcosine and 0.02% sodium dodecyl sulfate, Sigma-Aldrich^®^). Each slide was incubated overnight at 37°C under a plastic coverslip in a humidified chamber. The slides were then washed, and excess probe was removed by washing in increasingly stringent solutions consisting of 1 × SSC (saline sodium citrate) and 0.1 × SSC for 10 min at 42°C. To detect hybridization, 100 μl of StrepAvidin conjugated to peroxidase was applied to each slide, and the slides were incubated at 37°C for 1 h. The substrate as well as the procedures for counterstaining and slide mounting was the same as described for the IHC protocol.

### Scoring of immunohistochemistry and statistical analysis

The following statistical analyses were performed: 1) chi-square for independence for cytoplasmatic and non-cytoplasmatic immunoreactivity of AOP-1, MMP-9, SNAP-25 and US9 BoHV-5 ISH stain scored on a four-point scale from no reactivity (1) mild reactivity (2), moderate reactivity (3) intense reactivity (4); 2) Mann-Whitney U test for immunohistochemical results and anatomical areas presenting microscopic lesions. The images were taken using a light Axio Imager A.1 microscope connected to an AxioCam MRc (Carl Zeiss Oberkochen, Germany). The micrographs were processed with Axiovision 4.7 software (Carl Zeiss). For each slide, at least 7 microscopic fields were randomly chosen, and the mean number of positive signals was calculated and computed as number of reactive cells/mm^2^. The SAS v 8.2 (SAS Institute Inc. Cary, NC, USA) and Origin software were used for data analysis. P < 0.005 was considered as statistically significant.

### Ethical concerns

All animal handling and sample collection procedures were performed in accordance with the recommendations of the Brazilian College on Animal Experimentation (COBEA), and all experiments were approved by Institution Ethics and Animal Welfare Committee.

## Results

### Histopathology findings

**Figure 1:**
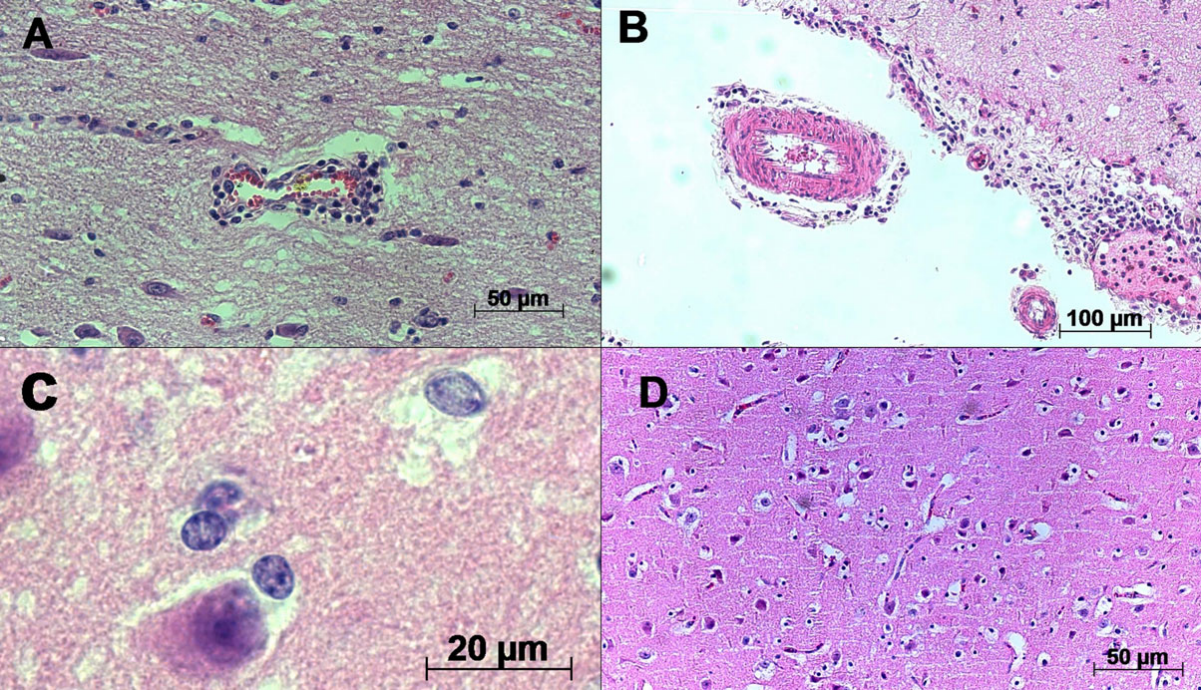
**Photomicrography of microscopic aspects of BoHV-5 infected calf brains**. A) Inflammatory cuffs composed of mononuclear cells in the parenchyma and B) in the leptomeninges; C) areas presenting inclusion bodies infrequently detected, D) satellitosis and gliosis distributed in the parenchyma and close to neurons. Hematoxylin & Eosin stain; scale bar 50 μm.

Brain lesions were mainly confined to the frontal cortex (Fc) and the parietal cortex (Pc) and were characterized as softening of parenchymal tissue and hemorrhagic foci in the Fc and Pc. The lesions were not symmetrical and lacked a well-defined pattern of distribution. No macroscopic lesions were seen in the CNS of the control group. In all cases, microscopic examination revealed histological changes of varying severity in several areas of the brain, but mostly in the Fc, Pc and thalamus. These changes were characterized by inflammatory cuffing, satellitosis, focal and diffuse gliosis and, rarely, inclusion bodies (Fig. 1B, C and 1D). An intense neuro-inflammation was described in all analyzed areas; however, it was almost exclusively seen in the Fc, Pc and thalamus (T) with a correlation coefficient of *r *= 0.8976. In most cases, the number of inflammatory cuffs was considered ≥ 70% and was considered statistically significant when compared to satellitosis (*p *= 0.0004). The likelihood ratio test yielded significant differences (*p*-value < 0.005) for gliosis, satellitosis and inflammatory cuffing between two groups (BoHV-5 infection versus healthy cattle). Inclusion bodies, however, were not frequently observed. The microscopic analysis performed in all cases provided confirmation that all animals were suffering from acute BoHV-5 encephalitis. In all negative samples studied, neither macroscopic nor microscopic alterations could be observed.

### IHC and ISH results

**Figure 2:**
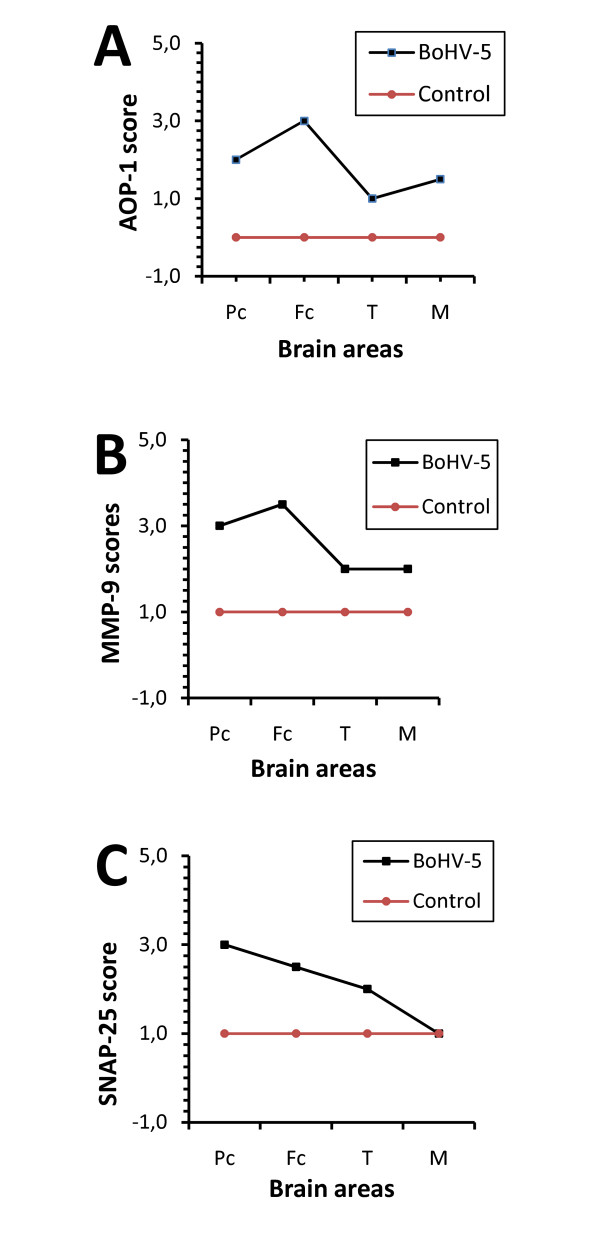
**Frequency of detection of antigens**. AOP-1 (A), MMP-9 (B) and SNAP-25 (C) proteins in different brain areas (Pc, Fc, T and M; x-axis) of naturally infected calves (n = 20) and a control group scored on a scale of 1 to 4.

**Figure 3:**
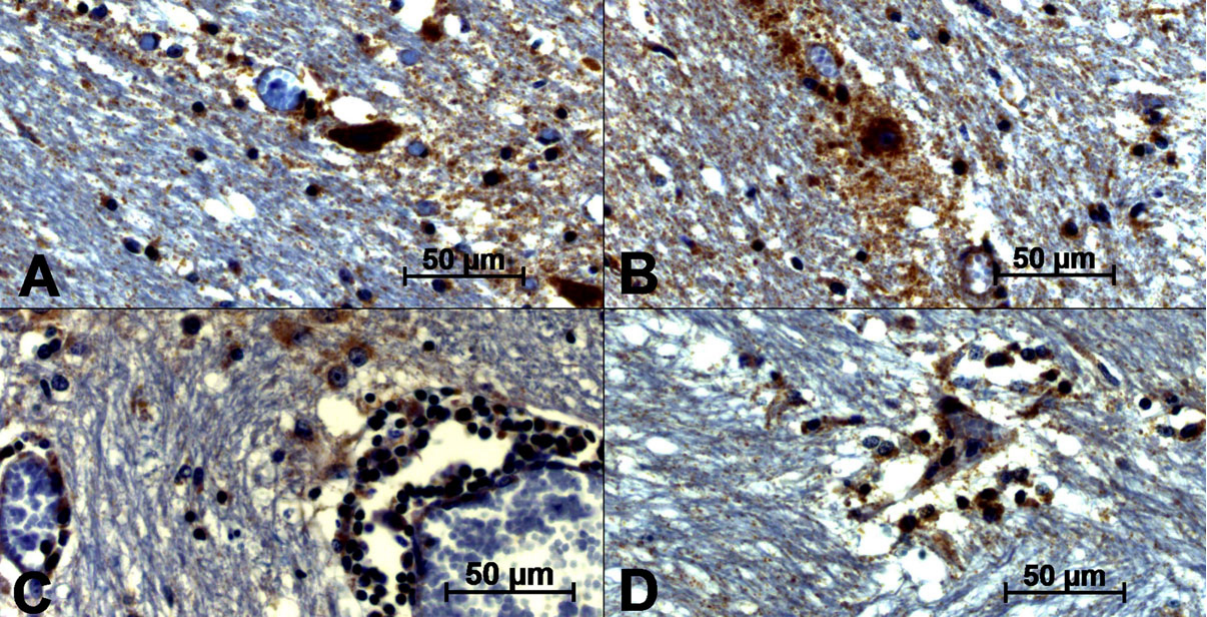
**Photomicrography of AOP-1 antigen detection by immunohistochemistry in the frontal cortex of BoHV-5-infected calves (A-D)**. A) area of intense immunoreactions in the parenchyma; B) intense positive signals surrounding neuron as well as the area of neuron; C) inflammatory cuffs showing intense positive reaction close to mononuclear cells; D) positive labeling associated with gliosis and satellitosis. The strepavidin-biotin peroxidase complex method was used. (scale bar 50 μm)

**Figure 4:**
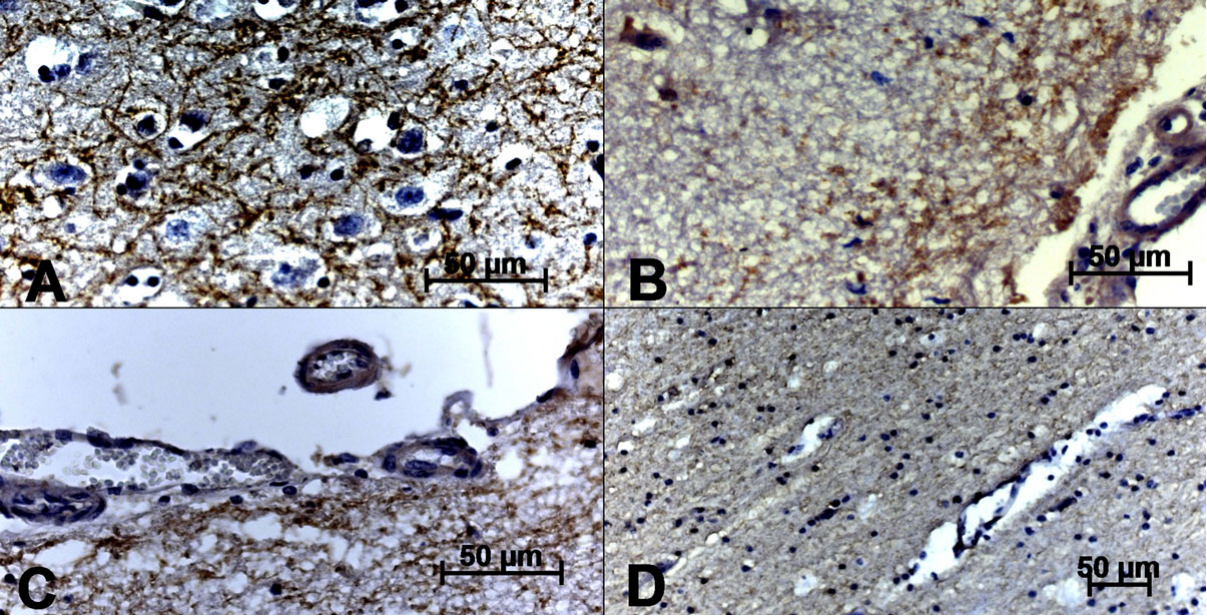
**Photomicrography of immunohistochemical MMP-9 antigen detection in the frontal cortex of BoHV-5-infected calves (A-D)**. A) area of intense immunoreactions in the parenchyma; B-C) intense positive signals in the subependimal zones; D) no positive labeling was associated to control group. The strepavidin-biotin peroxidase complex method was used (scale bar 50 μm).

**Figure 5:**
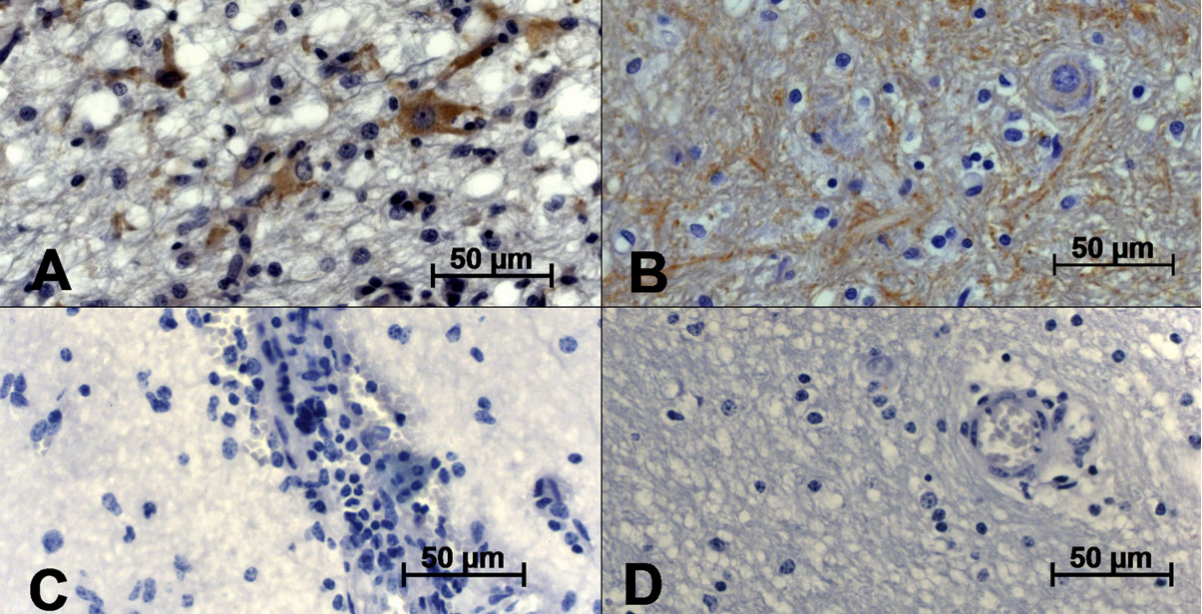
**Photomicrography of immunohistochemical SNAP-25 antigen detection in the frontal cortex of BoHV-5-infected calves (A-B) and control group (C-D)**. A) positive immunolabeling of neuronal and glial cells; B) positive labeling dispersed in the parenchyma; C and D) no positive reaction in the control group. The strepavidin-biotin peroxidase complex method was used (scale bar 50 μm).

**Figure 6:**
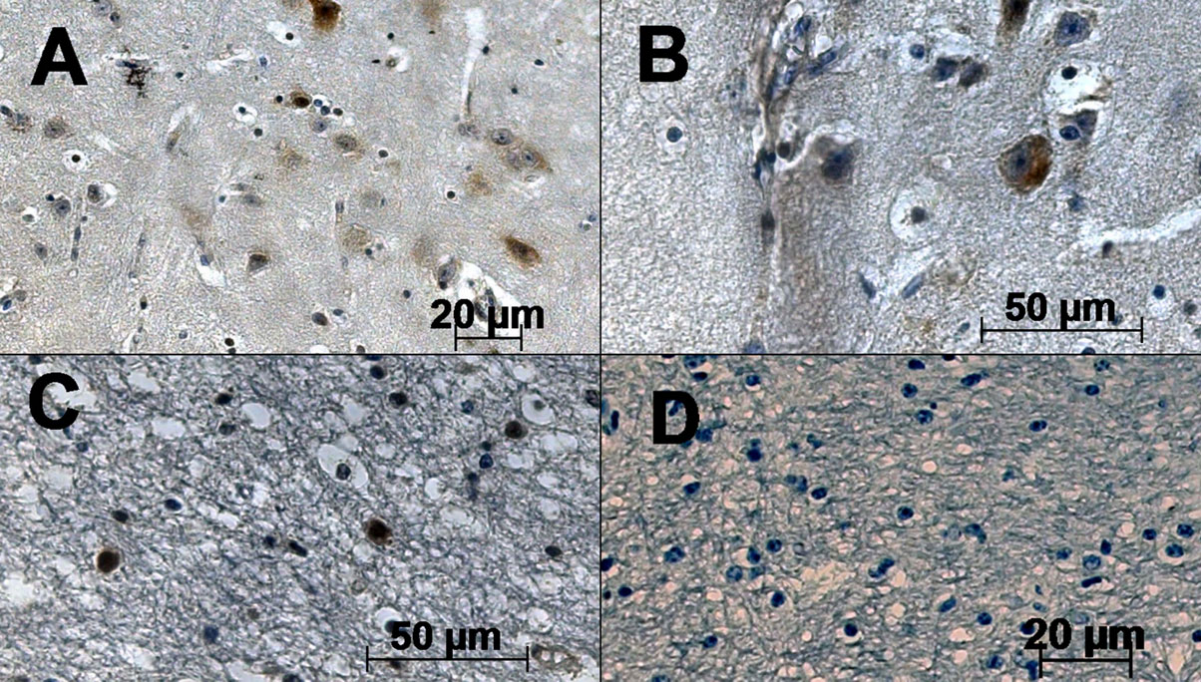
**Photomicrography of in situ hybridization assay**. A) positive detection of US9 BoHV-5 gene in brain cells; B) positive reaction in neurons; C) positive reaction in glial cells; D) control group. The strepavidin-biotin peroxidase complex method was used (scale bars 20 and 50 μm).

We observed a positive correlation (*r *= 0.8976) among AOP-1 (Fig. 2A), MMP-9 (Fig. 2B) and SNAP-25 (Fig. 2C) detected in all slides from the frontal cortex region. The AOP-1 staining was observed diffusely in the neuropil and in different types of brain cells, such as neurons, endothelial cells and glial cells (Fig. 3A-D). The MMP-9 staining was intensively visualized in the parenchyma and the cortical area of the Fc. Furthermore, the pattern of MMP-9 labeling was very similar to that of AOP-1 staining (Fig. 3A) and mostly visualized around neurons, endothelial cells, the subependymal zone and inflammatory cuffs (Fig. 4A-D). No unspecific label could be visualized in the control group. An intense change in SNAP-25 immune-labeling patterns was observed when comparing BoHV-5 and control cases (Fig 5A and B). The positive signals were mainly documented around neurons and microglial cells, and no signal was observed in the control group (Fig. 5C and 5D). Regarding to the US9 BoHV-5 gene *in situ *hybridization, positive cells were visualized mostly in Fc as shown in Fig 6A and 6B.

## Discussion

All animals included in the present study were field BoHV-5 cases selected by routine service in the Veterinary School, São Paulo State, Brazil. All symptoms were mainly described as loss of coordination and paralysis. Additionally, BoHV-5 infection was confirmed by virus isolation and molecular analysis [9,15,16]. The respective brain areas chosen in this investigation were based on positive results of *in situ *PCR amplification of the US9 gene of the BoHV-5 strain reported previously [16]. Although the olfactory bulb has been described as the principal brain entrance for BoHV-5 infection, no immunoreactions were visualized for the AOP-1, MMP-9 and SNAP-25 proteins in this portion [15,17]. As a consequence, only the frontal cortex, parietal cortex, thalamus and mesencephalon regions were considered for the analysis. Moreover, the use of natural cases of BoHV-5 infections is useful to address some questions on the cell signaling during immune response. Although experimental BoHV-5 infections of calves have been performed [1,5,10] the participation of oxidative stress, the integrity of the blood-brain barrier and synapses participation still need to be fully elucidated.

One of the most distinctive viral encephalitis lesions, characterized as inflammatory cuffs, satellitosis and gliosis, demonstrated good correlation with BoHV-5 US9 gene hybridization [15,17] Similar lesions have been previously demonstrated in acutely infected calves [3,6,10] and rabbits [7]. The inclusion bodies were rarely noted. Although this result is in contrast with most reports describing cases of BoHV-5 inclusions [1,8], one study has reported the absence of inclusions bodies in calves experimentally infected with BoHV-5 [3]..

Supporting the premise that BoHV-5 entry into the CNS is facilitated by leukocytes, like for other herpesviruses, we also suspect that virus infections activate the AOP-1, MMP-9 and SNAP-25 proteins in the inflammatory infiltrates, which has been described recently as being composed by CD3 + T lymphocytes [17]. Association between MMP-9 and herpesviruses has already been reported [11,18,19]. In vitro studies suggested that MMP-9 might contribute to Epstein-Barr virus (human herpesvirus type 4) spread [18], and the continued presence of HSV-1 as well as HSV-1 immune complexes is able to promote MMP-9 production [19]. However, the association of MMP-9 with AOP-1 and SNAP-25 expression related to BoHV-5 neurological cases has never been described in association with herpesvirus infections.

Living organisms produce reactive oxygen species during physiological processes and in response to external stimuli. To protect themselves against oxidative attacks as well as maintain a redox balance in their different subcellular compartments, cells have evolved complex mechanisms [20]. These antioxidant defense systems include non-enzymatic substances, such as vitamin E, vitamin C, vitamin A and uric acid, and also enzymes with antioxidant properties, such as catalase, superoxide dismutase and glutathione peroxidase, as well as low-molecular-weight reducing agents, including glutathione and thioredoxin. A wide variety of stimuli, including tumor necrosis factors (TNF-α), phorbol ester, bacterial lipopolysaccharide and viral infection, can activate the cellular immune response [14]. The antioxidant-like protein 1, (AOP-1, also called MER5, M.W. 25 kDa) a member of the family of proteins involved in defense against oxidative stress, seems to be involved in proliferation and differentiation, through anti-oxidant function and redox regulation [14]. In this study, the AOP-1 positive reaction mainly around neurons suggested that this phenomenon is involved in the host-BoHV-5 interaction. Unfortunately, no information is available on anti-oxidative mechanism related to neurological disorders caused by virus infections. However, AOP-1 production has been described in ischemic disorders involving the CNS [14].

Synaptic protein disorders have been related to clinical manifestations in a member of human and animal prionopathies [21]. This phenomenon is not surprising since prion proteins are closely associated with synaptic machinery [21]. In addition, SNAP-25 was intensively expressed, mainly in glial cells and neurons, in all the BoHV-5 cases studied. The biggest difference is the disease evolution; although prionopathies are characterized as degenerative disorders, the BoHV-5 cases studied here were classified as acute viral encephalitis [15].

In summary, the neuro-pathogenesis studies carried out on filed cases of BoHV-5 infections suggest that cellular immune responses, particularly those involving blood-brain barrier integrity, oxidative stress and neurotransmission, are of great significance in meningoencepahalitis cases related to BoHV-5 infection. However, further studies must be carried out to determine the implications of these events for the different stages of BoHV-5 replication (primary infection, latency and viral reactivation) in neurological disorders. The data provide a basis for further study of the role of these proteins that underlie the pathologic process in BoHV-5 encephalitis.

## Competing interests

The authors declare that they have no competing interests.

## Authors' contributions

TC, HFF, AFG and ALA carried out the practical work, performed necropsies, histological analysis and immunohistochemical assays. LCB and AHCN were mainly responsible for performing the statistical analysis. All authors read and approved the final manuscript.

## References

[B1] Belknap EB Collins JK Ayers VK Schulthesis PC Experimental infection of neonatal calves with neurovirulent bovine herpesvirus type 5 (BHV-5) Vet Pathol 1994 31 358 365 10.1177/030098589403100309 8053131

[B2] Carrillo BJ Ambrogi A Schudel AA Vasquez M Dahme E Pospichil A Meningoencephalitis caused by an IBR virus in calves in Argentina J Vet Med B 1983 30 327 332 10.1111/j.1439-0450.1983.tb01852.x 6310913

[B3] Bretschheider G Leunda MR Osorio FA Flores EF Odeón AC Primary infection, latency, and reactivation of Bovine Herpesvirus type 5 in bovine nervous system Vet Patho 2002 39 437 444 10.1354/vp.39-4-437 12126146

[B4] Diel DG Almeida SR Brum MCS Dezengrini R Wieben R Flores EF Acute and latent infection by bovine herpesvirus type 5 in experimentally infected goats Vet Microbiol 2007 15 257 267 10.1016/j.vetmic.2006.12.019 17267142

[B5] Flôres FS Flôres V Caron L Flores EF Weiben R Winkelmann ER Mayer SV Bastos RG Distribution of Bovine Herpesvirus type 5 DNA in the central nervous systems of latently experimentally infected calves J Clin Microbiol 2003 41 4512 4520 10.1128/JCM.41.10.4512-4520.2003 14532175 PMC294956

[B6] Vogel FSF Caron L Flores EF Distribution of bovine herpesvirus type 5 in the central nervous systems of latently, experimentally infected calves J Clin Virol 2003 77 10339 10341 10.1128/JCM.41.10.4512-4520.2003 PMC294956 14532175

[B7] Chowdhury SI Lee BJ Moiser D Sur JH Osorio FA Kennedy G Weiss ML Neuropathology of bovine herpesvirus type 5 (BHV-5) meningo-encephalitis in a rabbit seizure model J Comp Pathol 1997 117 295 310 10.1016/S0021-9975(97)80078-3 9502267

[B8] Silva AM Weiblen R Irigoyen LF Roeche PM Sur H-J Osorio FA Flores EF Experimental infection of a sheep with bovine herpesvirus type-5 (BoHV-5) Vet Microbiol 1999 66 89 99 10.1016/S0378-1135(98)00305-8 10227471

[B9] Cardoso TC Gomes DE Ferrari HF Silva-Frade C Rosa ACG Andrade AL Luvizotto MCR A novel in situ polymerase chain reaction hybridisation assay for the direct detection of bovine herpesvirus type 5 in formalin-fixed, paraffin-embedded tissues J Virol Meth 2010 163 509 512 10.1016/j.jviromet.2009.11.013 19917316

[B10] Meyer G Lemaire M Ros C Belák K Gabriel A Cassart D Coignoul E Belák S Thiry E Comparative pathogenesis of acute and latent infections of calves with bovine herpesvirus types 1 and 5 Arch Virol 2001 146 633 652 10.1007/s007050170136 11402854

[B11] Candelario-Jalil E Yang Y Rosenberg GA Diverse roles of matrix metalloproteinases and tissue inhibitors of metalloproteinases in neuroinflammation and cerebral ischemia Neuroscience 2009 158 983 994 10.1016/j.neuroscience.2008.06.025 18621108 PMC3584171

[B12] Leppert D Waubant E Galardy R Bunnet NW Hauser SL T cell gelatinases mediate basement membrane transmigration in vitro J Immunol 1995 154 4379 4389 7722295

[B13] Wang P Dai J Bai F Kong K-F Wong SJ Montgomery RR, Madri JA, Fikrig E: Matrix metalloproteinase 9 facilitates West Nile Virus entry into the brain J Virol 2006 82 8978 8985 10.1128/JVI.00314-08 PMC2546894 18632868

[B14] Hwang IK Hua L Yoo Ki-Y Kim DW Kang T-Ch Choi SY Won MH Kim D-H Anti-oxidant-like protein 1 is altered in non-pyramidal cells and expressed in astrocytes in gerbil hippocampal CA1 region after transient forebrain ischemia Brain Res 2005 1002 111 119 10.1016/j.brainres.2005.09.022 16256080

[B15] Ferrari HF Luvizotto MCR Rahal P Cardoso TC Detection of bovine Herpesvirus type 5 in formalin-fixed, paraffin-embedded bovine brain by PCR: a useful adjunct to conventional tissue-based diagnostic test of bovine encephalitis J Virol Meth 2007 146 335 340 10.1016/j.jviromet.2007.07.018 17804088

[B16] Cardoso TC Ferrari HF Luvizotto MCR Arns CW Bio-safety technology in production of bovine herpesvirus type 5 (BoHV-5) using an alternative serum-free medium Am J Biochem Biotechnol 2007 3 125 130 10.3844/ajbbsp.2007.125.130

[B17] Gomes DE Ferrari HF Roncatti FTB Paes F Cardoso LS Perri SHV Nogueira AHC Luvizotto MCR Cardoso TC Astrocyte intermediate filaments are important markers in olfactory bulb of bovine *Herpesvirus *type 5 natural infections Braz J Vet Pathol 2010 3 17 23

[B18] Flavell JR Baumforth KRN Williams DM Lukesova M Madarova J Noskova V Lowe D Murray PG Nelson PN Expression of the matrix metalloproteinase 9 in Hodgkin's disease is independent of EBV status Mol Pathol 2009 53 145 149 10.1136/mp.53.3.145 PMC1186921 10897334

[B19] Hayashi K Hooper LC Detrick B Hooks JJ HSV immune complex (HSV-IgG:IG) and HSV-DNA elicit the production of angiogenic factor VEGF and MMP-9 Arch Virol 2009 154 219 226 10.1007/s00705-008-0303-7 19115032

[B20] Saadoun D Bieche I Authier F-J Laurendeau I Jamboou F Piette JC Vidaud M Maisonobe T Cacoub P Role of matrix metalloproteinase, proinflammatory cytokines and oxidative stress-derived molecules in Hepatitis C virus-associated mixed cryoglobulemia vacuities neuropathy Arthritis Rheumatism 2007 56 1315 1324 10.1002/art.22456 17393409

[B21] Vidal E Márquez M Tortosa R Costa C Serafín A Pumarola M Immunohistochemical approach to the pathogenesis of bovine spongiforme encephalopathy in its early stages J Virol Meth 2006 134 15 29 10.1016/j.jviromet.2005.11.010 16406559

